# Understanding preservice teachers' affective responses to VR-enabled scientific experiments

**DOI:** 10.3389/fpsyg.2022.929270

**Published:** 2022-08-05

**Authors:** Tao Xie, Ling Zhang, Geping Liu

**Affiliations:** Faculty of Education, Southwest University, Chongqing, China

**Keywords:** scientific experiment, affective state, affective design model, virtual reality, educational technology, preservice teachers

## Abstract

Preservice teachers' preparedness, perception, and affect toward certain technology systems influence the student acquisition of science knowledge, process skills, teaching innovation, and willingness to use technology in their classroom. The purpose of this study was to explore teachers' affective responses to a virtual reality-enabled scientific experiment (VaSE) system. Fifty-one preservice teachers majoring in educational technology participated in the study. They were divided into two groups, and their reactions were measured separately on two occasions. The first occasion used a standard system following the principle of Donald Norman's affective design model (ADM). The VaSE system was then revised with a strict version of ADM before the participants' reactions were measured for a second time. The positive and negative affect scale (PANAS) was utilized for affective state evaluation. Semi-structured interviews that focused on affective experiences were organized using an ADM framework and conducted immediately after the participants used VaSE. The results indicated that the positive affect experienced by the preservice teachers was significantly enhanced, and the negative affect was significantly weakened. Academic level, gender, and prior experience were important random effect factors that impacted the affective experience. It was also revealed that participants were more likely to be affected by immersion and interactivity in terms of enhancing positive affect and were more likely to be affected by behavioral layer elements in terms of weakening negative affect. A conclusion has been drawn to provide theoretical and practical suggestions for training preservice teachers in ways that improve their ability to integrate technology into the classroom.

## Introduction

### Scientific experiments

A science-based curriculum encompasses a range of disciplines, including physics, chemistry, life science, etc. The goals of a science-based curriculum center on helping students acquire scientific knowledge and cultivate their critical thinking skills and practical abilities (Fauth et al., 2019). Scientific experiments are one of the most important parts of science curriculums because they give students opportunities to use specific instruments and equipment while observing and studying scientific phenomena and regularities through artificially controlled conditions. Experiments that take place in the early stages of a student's education typically focus on the acquisition of scientific terms, concepts, and facts. Teachers provide students with detailed explanations about the experimental process, phenomena, and experience in advance (Concannon and Brown, 2008), and students carry out scientific experiments in a step-by-step manner with predetermined results. Although scientific experiments are time-effective for traditional classrooms, students focus on developing an understanding of experimental manuals and accurately performing each of the required steps. It can be difficult for them to associate the content of experiments with real life, and they are less likely to develop high-order cognitive skills using these methods (Madhuri et al., 2012). In more recent decades, the goal of scientific experiments has shifted to using technology to make scientific findings, solve problems, and support students in ways that allow them to understand scientific phenomena and identify the steps involved in experiments like scientists (Ahmed and Parsons, 2013). Many scholars have thus claimed that employing proper technology in scientific experiments promotes better learning motivation, project performance, critical thinking, and achievement scores (Chang and Hwang, 2018; Lamb et al., 2018).

### How VR enables scientific experiments

Scholars have begun to consider how virtual reality (VR) can be used to display scientific experimental scenarios in science education. Lamb et al. (2018) investigated the difference between using VR and hands-on activities during scientific experiments and concluded that there was parity between the conditions in terms of learning outcomes and cognitive processing. In other words, VR can address the limitations of hands-on activities that do not take place in a real laboratory. Liu et al. (2020) conducted quasi-experimental research and concluded that students who were exposed to VR-enabled science lessons tended to have higher levels of academic achievement and engagement than those who learned in traditional science classrooms. Most scholars believe that the successful use of VR in classrooms encourages students to fully explore the scientific world without worrying about causing harm (Moro et al., 2017; Liu et al., 2020). Students who are exposed to VR learning environments are able to observe experimental phenomena that cannot be encountered in the real world (Artun et al., 2020). Examples of VR usage include presentation and simulation of cell division, internal molecular structures, and visual changes in magnetic fields. Scholars have recommended that universities and schools use VR platforms due to their unique properties (i.e., immersion, interaction, and imagination) (Concannon et al., 2019).

VR-enabled scientific experiments (VaSE) are grounded in a computer-generated simulation environment that embodies abstract scientific concepts, presents complex information in an easy-to-understand manner, and displays experimental phenomena that cannot be directly observed (Artun et al., 2020). A recent study reports that VaSE has a positive impact on the academic performance and attitudes of individual students in science courses (Sahin and Yilmaz, 2020). VaSE is considered to be an interactive learning environment that effectively reduces the impact of material unevenness, insufficient equipment, and sudden weather changes that are prominent in traditional experiments. The usefulness of VR in scientific experiments is also reflected in its increasingly important cognitive foundation. Lamb et al. (2018) believe that the next generation of science education standards will emphasize the role of cognitive strategies in science courses. The development of cognitive strategies is an important step toward understanding the learning processes and results of students. The cognitive system is related to specific stimuli and activation intensity. The patterns of information presentation in VaSE will adjust students' cognitive needs and dynamics, which is conducive to critical thinking and modal representation.

### Why preservice teachers' affect is focused

Because the success of students during scientific experiments is dependent on their teacher's ability to integrate technology into the class, there is a great demand for highly qualified professionals to guide scientific experiments that use VR. Artun et al. (2020) stated that it was equivalently important for the training of preservice teachers and school students in terms of acquisition of science knowledge and skills. However, most teachers struggle to use technology or do not use it in meaningful ways despite the fact that adequate equipment and funds are invested in scientific laboratories (Atabek, 2020). Therefore, many universities in China have teacher education programs for preservice teachers that focus on the theoretical and practical aspects of integrating technology into the classroom. With the growing emphasis that is being placed on the next generation of science standards, preservice teachers need to prepare themselves by developing decision-making skills and learning how to implement pedagogies in a range of educational scenarios (Lamb and Etopio, 2020). Preservice teachers are required to develop digital competencies and positive attitudes toward the technology that they will use. Preservice teachers' ability to use VaSE and their attitudes toward this technology largely determine their behavioral intention to use this system in their future careers (Farjon et al., 2019). A teacher's attitude toward technology has cognitive, skill-oriented, and affective aspects and is a learned predisposition that influences how they will respond to certain objects, situations, or behaviors (Suárez et al., 2019). A number of publications have concluded that preservice teachers' attitudes toward technology significantly affect their ability and intention to adopt the system in class (Baturay et al., 2017; Gavaldon and McGarr, 2019). Avsec and Jagiełło-Kowalczyk (2018) have claimed that preservice teachers' attitudes toward technology are related to the feelings that they experience when they are first exposed to it. In short, a positive attitude toward technology is linked to increased usage and teaching engagement for preservice teachers.

Although scholars have investigated preservice teachers' attitudes toward technology-enhanced learning, not many publications have focused on the domain of attitude (i.e., affective aspect). Scholars have claimed that barriers to technology integration in class include teaching beliefs and attitudes toward using technology, but they have also suggested that research should pay special attention to the affective aspects of attitudes (Beri and Sharma, 2019). The affect typically involves both physiological and cognitive processes. Many scholars utilize a circumplex model of affect to identify the relative relationships among different sets of affective states ranging from the constructs of the pleasant–unpleasant continuum and aroused–unaroused continuum (Dozio et al., 2022). Students with specific affective states can develop a sense of emotional well-being that influences their behavior and cognition. Affect also plays a critical role in the learning process. Positive affect is more likely to stimulate internal learning motivation, improve enthusiastic participation, reduce the perceived difficulty of learning materials, and promote knowledge retention and learning transfer (Wong and Adesope, 2021). Research has indicated that the high-quality emotional design of a learning environment can induce positive affects and improve learning effectiveness, task performance, and the level of pleasure and satisfaction involved in the learning experience (Shangguan et al., 2020). Many parameters of learning environments are closely related to the affective states of students, for example, brightness, simulated weather conditions, colors, and geometric renderings can elicit joy, sadness, boredom, anxiety, and relaxation. Studies have claimed that replicating real environments in virtual environments can also incite the affective responses of students because it is easier to relate to real situations through a sense of embodiment (Barbot and Kaufman, 2020).

The affect is composed of positive and negative dimensions, triggered by the subjective judgment of an individual's response to teaching materials and environment. Affect has a significant impact on physiological and psychological status when it comes to decision-making, perception, and learning (Marín-Morales et al., 2020). Numerous studies have thus referred to affective computing or affective modeling (Makransky and Lilleholt, 2018; Yadegaridehkordi et al., 2019). Positive affect enhances teaching motivation, beliefs, technology adoption, and class performance and is also related to specific cognitive processes, such as information processing, communication processes, negotiation processes, decision management, classification tasks, and creative activity settings (Um et al., 2012), while negative affect acts in the opposite way. Positive affect makes individuals select effective information fragments from their long-term memory and combine them with previous knowledge (Mayer and Estrella, 2014). Research suggests that individuals with lower levels of prior knowledge are better able to obtain positive affects from compassionate and encouraging affective design elements (e.g., pedagogical animated agents) (D'mello and Graesser, 2010), while individuals with high levels of prior knowledge have the opposite experience. Because preservice teachers with specific affective states have tremendous potential to pass their values on to students, assessing the affective responses that preservice teachers have to the VaSE system can provide useful insights into integration optimization and strategic use with respect to technology.

Another reason why this study highlights the affective aspect rather than attitude *per se* is that affect has an important moderating effect on attitude in view of individuals' cognitive processing. Cognitive load theory divides cognitive load into endogenous cognitive load and exogenous cognitive load (Amadieu et al., 2009). The endogenous cognitive load results from the complexity of tasks relative to an individual's prior knowledge and strategy use, while the exogenous cognitive load originates from the sub-optimal design of the learning materials and environment and requires the additional filtering of information that makes no sense to the learning task (Schneider et al., 2016; Brom et al., 2018). In order to counteract the extent to which the exogenous cognitive load consumes cognitive resources, the affective design model (ADM) of the learning environment can be used to activate deeper cognitive processing and effectively enhance the individual's affective efficacy. The ADM matches the presentation and interaction of information with the learning environment, which uses different design elements (i.e., animation, interesting text, audio, video, etc.) to influence individual experiences and enhance affective efficacy (Plass and Kaplan, 2016). Triberti et al. (2017) believe that affect is a cognitive process that plays an important role in the design of interactive learning environments. In this context, affect mediates between thinking ability, decision-making, attention, memory, and meaning construction (Blanchette and Richards, 2010).

### Aim of this study

In summary, preservice teachers play a critical role in helping students develop scientific knowledge, process skills, and affective values in a technology-integrated experimental curriculum (Artun et al., 2020). Although some studies have reported that technology-innovated education is important for preservice teachers, few publications have profoundly emphasized the use of VaSE for the training of preservice teachers. According to a recent systematic review, the use of VaSE for educational contexts appears to be in its infancy, especially in the domain of subject education (Ade-Ojo et al., 2022). Recently, VaSE has been used to improve the affective achievement of preservice teachers (Han et al., 2022). However, studies that have examined the impact that VR technology has on preservice teachers' affect focused on empathetic reactions and did not integrate the effective ADM into their framework. There are also some studies that examine the impact that preservice teachers' attitudes toward VaSE have on their motivation and behavioral intention, but not much work has been done that focuses on the domain of attitude (i.e., affective aspect). Huang et al. (2022) have stated that the negative affect that teachers experience when using technology in learning environments is connected to job-related stress, which can cause negative physical, psychological, and vocational consequences for preservice teachers and impact their teaching success. Because of this, preservice teachers' affective responses to VR learning environments may also determine students' learning beliefs, performance, and academic achievements. Therefore, the aim of this study was t to explore preservice teachers' affective responses to VaSE through the integration of ADM. We provide theoretical and practical suggestions for how preservice teacher training can improve the ability of teachers to integrate technology into the classroom. In particular, this study addresses the following three questions:

(1) Do preservice teachers experience a predominantly positive or negative affect while using VaSE?
(2) Is the affective experience of preservice teachers significantly improved after the use of VaSE?
(3) What affective design elements influence the experiences of preservice teachers?

## Materials and methods

### System configurations

**Figure 1:**
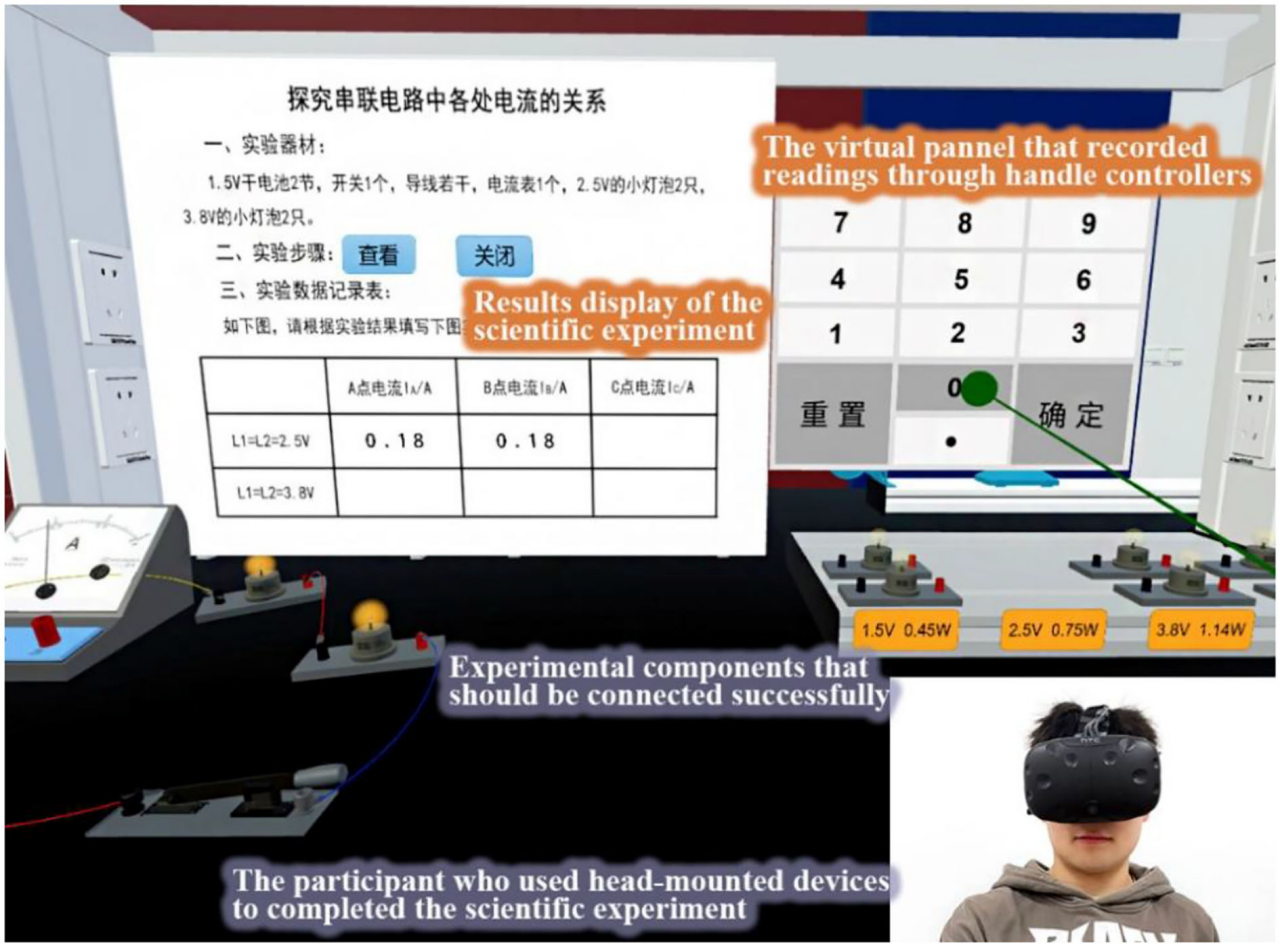
The developed VaSE system.

The VaSE system used in the current study was a self-developed VR application built around the theme Exploring the Relationship between Currents in Series Circuit (see Figure 1), which corresponds with the first lesson in most physics textbooks at the junior high level in China. The system was designed to train preservice teachers to integrate VR into the classroom so that they could help students explore the properties of electric current and connect elements more effectively. The preservice teachers' affective responses to VaSE were first investigated as an important part of the training because their affective experiences were directly connected to their attitudes and intention to use the VaSE system and influenced the innovative practice of teaching. For this purpose, preservice teachers used the VaSE system in their training course and were required to connect the ammeter, light bulbs, batteries, switches, and wires and record corresponding readings on the ammeter by selecting digits on a virtual panel once the light bulbs were successfully lit. Then, they were expected to report their affective responses to the VaSE system.

The hardware devices used for this study consisted of a group of HTC VIVI kits. Each kit included a high-performance computer with a high-definition screen, a wired head-mounted device (HMD) with a resolution of 2160^*^1200, two handle controllers, and a pair of laser positioners. The software compiling VaSE was a VR editor called VeryEngine. The VeryEngine, structured on the popular game engine Unity 3D, features Microsoft Excel-style editing functions so that it is easy to set parameters for virtual objects and define interactive behaviors between human and virtual objects.

Donald Norman's ADM (Norman, 2004) was used to guide the configurations of VaSE because it is well known for its effective design of technology and products. Based on the principles of cognitive science and product design, the ADM divides affect into visceral, behavioral, and reflective layers that correspond to three different levels of information processing (i.e., emotion, cognition, and reflective thinking). The visceral layer mainly considers the aesthetics of the VaSE system that dominate the human biological response and lead to rapid judgments about the system presentation (e.g., vision, hearing, touch, etc.). The behavioral layer focuses on the physical perception of the usability, effectiveness, and ease of use of VaSE and reflects the extent to which individuals actively use VaSE. The reflective layer is linked to higher-level cognitive processing, including learning satisfaction and perceived accomplishment, which maintains an individual's empathy for the VaSE system or related technologies to convey self-image and meaningful understanding to others.

To examine the effect of emotional design, this study developed two versions of VaSE. The VaSE used for occasion 1 was implemented for pure functionality and did not include the affective responses of participants to the design pipeline. Participants invited for occasion 1 were encouraged to report their perceptions about factors that lead to negative affects. Because most of the participants mentioned low fidelity of scene, difficulty in component connection, and high false touch rate, corresponding improvements were made by mapping the factors to the visceral, behavioral, and reflective layers and updating the VaSE for occasion 2. The configurations of VaSE used on the two different occasions are presented in Table 1.

**Table 1:** Two-occasion configurations of VaSE.

**ADM**	**Occasion 1**	**Occasion 2**
Visceral layer	Create tables, ammeters, light bulbs, batteries, switches, and wire models just as people do in a real laboratory.	Improve the reality of the models. Modify the *wires* so that the connected nodes are highlighted in red. When the student makes a mistake, the system prompts the student with voice feedback instead of text.
Behavioral layer	Participants are able to use handles to *pick up, roam, place, drop down item*s in VaSE.	Reduce the difficulty of circuit connection. The distance between endpoints within a predefined value will be automatically connected. Reduce the number of false touch operations (i.e., multiple selections and wrong selections).
Reflective layer	When connected correctly, the light bulbs will glow, and the readings of the ammeters will change; when the student fails to connect, the system will provide text feedback.	When the student has made no action with the experiment for more than 3 min, the system prompts them to complete the next operation; when the student makes 3 consecutive errors, the system explains the error and shows the correct operation to the student. When the student succeeds, the system will display an animated prompt (e.g., “Awesome” or “Great”).

### Experimental design

A combination of quantitative and qualitative methods was used in this experiment. In the quantitative research section, occasion 1 was a baseline assessment, and occasion 2 was an experimental assessment. The occasion-based configuration of VaSE was the independent variable, and the set of affective states of participants was the dependent variable. Participants' prior experience, gender, age, etc. were viewed as covariates. Qualitative research was conducted after participants finished the scientific experiment as a means of learning about the participants' affective experience.

### Participants

Participants for the study consisted of undergraduate students and postgraduate students who were majoring in education technology at our university in 2021. The participants had mastered corresponding instructional design skills and were willing to teach information technology courses in elementary and middle schools after graduation. They were identified as qualified preservice teachers for this study. Before the experiment, two research assistants conducted brief online or face-to-face interviews to ensure the legitimacy of the participants.

Fifty-one participants were divided into two groups and assigned to the two measuring occasions. Twenty-seven senior undergraduates participated during the first occasion. The number of male participants was 13, and the number of female participants was 14. There were 22 participants who had prior experience using the immerse VR system, and 5 participants who reported having no such previous experience. At the end of the first occasion, we revised the VaSE system according to the strict version of ADM. Three months later, the second occasion of the study took place. A total of 24 students, including 17 undergraduates and 7 postgraduates, participated in this phase of the study. There were 10 men and 14 women. Twenty-two participants had prior experience with using immerse VR system, and two participants reported having no such previous experience.

In order to account for potential differences in how well the participants remembered series circuits, the research assistant reviewed this concept and showed the participants the detailed operations of VR devices before the scientific experiment began. Each participant needed to connect circuit components and record readings through the virtual panel within 30 min. Right after they finished using the VaSE, participants filled out an affective state scale and answered a few questions about their affective experience in a semi-structured interview. Because the VaSE system was deployed in a single VR device, participants were required to participate in the experiment one at a time.

### Instruments

#### Affective state

The positive and negative affect scale (PANAS) developed by Watson et al. (1988) was used to measure the affective states of participants using the VaSE system. This scale contains two dimensions (i.e., positive affect [PA] and negative affect [NA]). Each dimension is described by 10 adjectives. Measures of PA include interested, attentive, excited, alert, enthusiastic, inspired, proud, determined, powerful, and active. Measures of NA include distressed, upset, hostile, angry, scared, afraid, ashamed, guilty, nervous, and jitter. There are 20 items in total, and each item was quantified by a Likert scale that ranged from 1 to 5, where 1 represents very inconsistent and 5 represents very consistent. Crawford and Henry (2004) have confirmed that both PA and NA scales have high validity and reliability. We revalidated the scale for the present samples and found that Cronbach's value of PA is 0.90 and Cronbach's value of NA is 0.85, which are consistent with the recommendations of Crawford and Henry (2004), indicating that the scale has good reliability for this study.

#### Affective experience

Under the framework of Donald Norman's ADM, a semi-structured interview form centered on affected experience was developed to investigate the participants' perception of VaSE. The instrument includes 10 items. Items 1–3 correspond to the affective experience at the visceral layer and examine the sensory preferences and affective responses of participants when they are exposed to VaSE. Items 4–6 correspond to the affective experience at the behavioral layer and examine the influence of the usability, ease of use, and controllability of the VaSE system on the affective experience of the participants. Items 7–9 correspond to the affective experience of the reflective layer, which examines the participants' sense of identity and satisfaction after using the VaSE system. In the last item, the participants described the overall feeling that they experienced while using the system.

### Data analysis

To address research question 1, this study calculated the mean and standard deviation for each affective dimension. If participants have higher scores with respect to positive affect and low scores with respect to negative affect, it is more likely that they had a better affective experience while using VaSE. To answer research question 2, we stacked the data in columns because each individual has multiple measures to represent different affective states. We then used a multi-level linear model in SPSS to test the significant changes in the comprehensive affective states of the participants. The measurements for each individual were processed at level 1, and measuring occasions were processed at level 2. Level 1 estimated within-individual changes using a linear model, and level 2 estimated between-individual differences after undergoing VaSE. Academic level, prior experience, and gender were treated as random effect variables. To answer research question 3, we conducted a text analysis by categorizing the interview data and summarizing similar responses in each category.

## Results

### Do preservice teachers experience a predominantly positive or negative affect while using VaSE?

The statistics of the affective scores of participants from both occasions are shown in Table 2. On the first occasion, participants had an average score of 3.93 for positive affective states with a standard deviation of 1.041 and an average score of 2.03 for negative affective states with a standard deviation of 1.107. On the second occasion, the participants had an average score of 4.12 for positive affective states and a standard deviation of 0.904. The average score for negative affective states was 1.68 with a standard deviation of 0.871. The results showed that compared to occasion 1, the positive affect of the participants on the second occasion increased, and the negative affects decreased. Both the scores for positive and negative affects tend to be concentrated. This indicates that the experiences of the preservice teachers produced a predominantly positive affect on both occasions. However, during occasion 2, this positive affect was even more pronounced and the negative affect was eliminated.

**Table 2:** Statistics of the affective states.

	**Occasion 1**		**Occasion 2**	
	**Average**	**Std**.	**Average**	**Std**.
PA	3.93	1.041	4.12	0.904
NA	2.03	1.107	1.68	0.871

### Would the affective experience of preservice teachers be significantly improved after the use of VaSE?

The results of the multilevel linear model are shown in Table 3. It is fairly clear that the changes in the positive (or negative) affective states of the participants tended to be significant after using the VaSE system during occasion 2. The positive affect produced during the first occasion was significantly lower than the positive affect produced during the second occasion (*t* = −5.104, *p* < 0.001), but the negative affect produced during the first occasion was significantly higher than the one produced during the second occasion (*t* = 4.972, *p* < 0.001). Academic grade and prior experience significantly affected the participants' positive affective experiences. Gender had no obvious influence on positive or negative affect. There was a significant interaction between academic level and prior experience and between academic level and gender. The influence of academic grades on negative affect was statistically significant, but the influence on negative affective experience was not obvious. This indicated that the affective experiences of the preservice teachers significantly improved after they used the strict version of Donald Norman's ADM.

**Table 3:** Results of the multilevel linear model.

	**PA**			**NA**		
	**Estimate**	**t**	**Sig**.	**Estimate**	**t**	**Sig**.
Intercept	7.663	11.827	0.000^**^	0.821	1.188	0.236
Occasion 1 vs. Occasion 2	−0.567	−5.104	0.000^**^	0.589	4.972	0.000^**^
Academic level	−1.125	−6.346	0.000^**^	0.329	1.733	0.084
Gender	−0.572	−1.897	0.058	−0.572	−1.780	0.076
Prior experience	−0.626	−4.074	0.000^**^	0.362	2.208	0.028^*^
Academic level * prior experience	0.194	4.481	0.000^**^	−0.122	−2.646	0.008^**^
Academic level ^*^ gender	0.246	2.701	0.007^**^	0.129	1.329	0.185

* Significant at p < 0.05,

** significant at p < 0.001.

### What were the affective elements that influenced the experience of the preservice teachers?

The factors that influenced the positive (negative) affective experience are shown in Table 4.

**Table 4:** Factors that linked to affective experience.

	**PA**			**NA**		
	**Factors**	**Count**	**Total**	**Factors**	**Count**	**Total**
Visceral layer	Strong Environmental immersion	13	22	The experimental equipment is floating in the air, and there is no sense of gravity	5	34
	high environmental reality	8		The “connected” status of the node is not obvious	9	
	beautiful environmental presentation	1		The experimental component is not within the first line of sight	2	
				The laboratory layout is complicated	3	
				“Wire” is low in realism	15	
Behavioral layer	Strong system interaction	10	16	Low accuracy of selecting experimental components and high false touch rate	17	39
	Complete system functions	3		Difficulty in circuit connection	18	
	Realization of operations that cannot be accessible in the real world	3		Complicated handle operation	2	
				The experimental components are difficult to control	2	
Reflective layer	No worry about the loss of experimental equipment	4	12	No guidance and feedback when the operation is difficult	1	9
	A sense of accomplishment and satisfaction	1		No obvious error prompt	7	
	Novelty	7		Weak time concept and low sense of security	1	

The visceral layer that led to the positive affect on the participants mainly included strong environmental immersion, high environmental reality, and beautiful environmental presentation. The behavioral layer that caused the positive affect on participants mainly included strong system interaction, complete system functions, and realization of operations that cannot be accessible in the real world. The factors that aroused positive affect in the reflective layer mainly included novelty, no worry about the loss of experimental equipment, and a sense of accomplishment and satisfaction. In view of the responses that were measured during the first occasion, both visceral layer elements and behavioral layer elements played an important role in stimulating participants' positive affect. It can also be inferred from the frequency of reflective layer elements that the optimization of this layer can improve participants' positive affective experience.

The visceral layer that caused a negative affect on participants included the low authenticity of experimental equipment, the lack of obvious experimental phenomena, and the complicated layout of the laboratory. In particular, in VaSE the wire cannot be bent, which made it more difficult to make a successful circuit connection. With respect to the behavioral layer, the factors that caused participants' negative affective experience included difficulty making a circuit connection, high false touch rate of components, complicated handle operation, and difficult control of experimental equipment. The reflective layer that caused negative affects for the participants included inconspicuous error prompts, lack of experimental help, weak conception of time, and insecurity.

Many participants believed that the sense of immersion and interaction provided by the learning environment played a major role in promoting their positive affect. For example, one participant made the following statement: “The scene and the experimental apparatus are almost real. I can easily complete all the experiments, and I really have a feeling of doing them in the laboratory.” The participant seemed to have been influenced by the immersive quality of the VaSE system. In this context, the word immersion describes the way that the participant was psychologically enveloped in the experience and is related to the extent to which the system's presentation (i.e., visual interface and interaction) replicates the real world. In this study, perceived immersion activated a type of psychological perception called presence, which created a sense of “being there” by means of the visible world and tangible interactions. Because the participant experienced reality, she determined that the system was usable and was able to become more engaged in the learning process.

Conversely, difficulty in operating the experiment apparatus was more likely to elicit a negative affect. One participant made the following remark: “It's hard to connect the wire ends to the binding posts, and I am not sure if the node is broken again due to my other operations.” When participants failed to accomplish the tasks in the experiments, they felt unable to use VR technology and ranked their ease of interaction poorly. The negative affect was mapped to the physical perception of the usability and behavioral intentions, influencing their judgments about the value of the VR system. It also damaged their belief in the effectiveness of VaSE because a sense of frustration impeded them from completing the experimental task.

## Discussion

### Findings

The findings of this study can be summarized as follows: (1) After exposure to VaSE with Donald Norman's ADM, the positive affect that the preservice teachers experienced was significantly enhanced, and the negative affect was significantly weakened. (2) Academic level, gender, and prior experience had varying degrees of influence on affective experience. (3) Among the factors that lead to the enhancement of positive affect, participants were more likely to be affected by the immersive and interactive components of VaSE, while among the factors that lead to the weakening of negative affect, participants are more likely to be affected by behavioral layer elements.

Using an optimized ADM could change the way that preservice teachers affectively experience VaSE in teacher education programs. A large number of studies support the idea that the visual design of the VR environment has a significant impact on individuals' affective social skills. This is because, for a considerable number of students, the intervention of teaching materials is mainly realized through interactive visual materials and the environment (Lorenzo et al., 2016). The findings of this study support empirical studies that argue that affect is an important medium for the effective interaction between students and VR learning environments. The affect may induce emotional, behavioral, and physiological responses to VaSE, leading to a series of positive changes in cognition, behavior, or reflection. The impact of positive affect on the learning process is mainly achieved by evoking stronger feelings of satisfaction, presence, and immersion within students. These feelings are closely related to the cognitive process and motivation to use the system and greatly influence changes in affective states. This inference, also identified by Riva et al. (2007) and Ip et al. (2018), explains why this study considered the visceral, behavioral, and reflective aspects separately based on Donald Norman's ADM. This study also used academic level, gender, and prior experience as random effect variables, which have varying degrees of impact on the affective experience that users feel in the VR environment. This result is basically consistent with most of the current literature. Preservice teachers' affective responses to high technical environments such as VR are potentially correlated with their technological knowledge about the environment. Gender differences could interact with both the subject content and the technical environment. Males and females differ with respect to various physiological and biological attributes, which can lead to different affective and cognitive consequences. Intuitively, female participants are less attracted to VR learning environments because they are more likely to experience a range of difficulties while operating within that environment, which could impede their positive affect and strengthen their negative affect. The findings, however, showed no significant gender differences in affective responses during the two occasions. This is partially because the participants were familiar with the subject content and encountered similar technical obstacles during their exposure to the system.

Although gender is not statistically significant in this study, the interaction between gender and academic level has a significant impact on the positive affective experience. That is to say, male participants with more academic experience tended to have a more positive affect during occasion 2, but this dynamic did not occur with the negative affect. It also seems clear that the academic level of participants was an important factor that influenced their positive affect. Academic level and its interactions with prior experience are linked to positive affect, but this rule did not apply to negative affect. This indicates that the postgraduate participants were more likely to recognize the immersion and interaction attributes of the system than the senior undergraduate participants. The impact of prior experience on positive affect is significant at the 0.001 level, and the impact on negative affect is significant at the 0.05 level. This indicates that Donald Norman's ADM embedded in VaSE significantly enhances the positive affect of preservice teachers and reduces negative affect. This finding is consistent with the work of D'mello and Graesser (2010), who believe that students with low prior knowledge are more likely to have a positive affective experience.

The findings revealed in this study differ significantly from the methods of current teacher training programs for experienced teachers. This study focuses more on the affective aspects of preservice teachers because the affective experiences of teacher candidates often have a more pronounced influence on their attitudes, motivation, and intention to use the system than educational content (Eutsler and Long, 2021). Preservice teachers make up a large proportion of educators in China and include not only normal students but also students who are willing to engage in education-related careers. Teacher education programs act as important buffers before preservice teachers become novice teachers. Chen (2022) believes that VR training experiences can improve the speed and effectiveness of the participants who are in the process of transferring the knowledge that they learned in school to an actual classroom setting. It is thus crucial to give preservice teachers as much VR experience as possible before sending them off to educate students in future classrooms. Most studies accept that using VR to train preservice teachers offers them low-stakes opportunities to practice developing professional skills and avoid making a potentially negative impact on the learning of actual students. However, they did not adequately explore how the affective design of VaSE influences preservice teachers' affective states and experiences. The findings of this study identify several empirical consequences in this context. After participating in the training involved in this study, all of the participants were able to successfully complete the circuit connections, which created positive changes in their affective states. This result suggests that our approach to educating preservice teachers through the integration of VR technologies in scientific experiments is effective. In the existing literature, preservice teachers' affective responses usually correlate with a willingness to use VR because affect is treated as an important component of attitude. Cooper et al. (2019) have observed that preservice teachers have a relatively high level of interest in using VR in their classrooms. Our results show that VaSE enhances positive aspects of affect, which leads to a greater chance that users will learn from VaSE and transfer their experience to real-life situations.

### Implications

The findings of this study indicate that preservice teachers should be taught how to integrate technology into their classrooms. With the rapid development of information technology, an important responsibility of higher education is to train highly qualified and well-equipped preservice teachers who are able to respond to the growing needs of society. Technology-integrated teaching has become increasingly prevalent in contemporary education because it is clear that technologies can improve class performance and higher-order thinking capacity if used properly. Traditional teacher training programs are not effective because preservice teachers do not have many opportunities to immerse themselves in different learning contexts (Ye et al., 2021). More importantly, traditional teacher training programs do not understand how preservice teachers use technology, which is the core component of digital literacy. The VaSE presented in this study offers an effective means of understanding preservice teachers' views and perceptions with respect to the affective aspects of integrating VR into science education. Previous scholars (Joo et al., 2018) have shown that the factors that influence preservice teachers' intention to use technology include teacher perceived usefulness, perceived ease of use, and self-efficacy, which partially correspond to the visceral, behavioral, and reflective layers of VaSE. Bower et al. (2020) have argued that the factors associated with preservice teachers' intention to use VR in class include effort expectancy, hedonic motivation, and behavioral intention, which can be mapped to internal- and design-related issues. Although these studies did not explicitly mention the impact of technology use on affective aspects, affect is a potential and implicit variable that mediates the acquisition of knowledge and the development of attitudes. Cheung and Vogel (2013) have suggested improving individuals' positive affective responses toward technology conditions so that they can actively use the technology to improve performance in organizations. Furthermore, using VaSE in teaching education programs also improves the reflective practices of preservice teachers by encouraging them to consider their affective states. McGarr (2021) has reported that virtual simulations provide preservice teachers with unique opportunities to experience examples of classroom life in a controlled manner, which thereby enhances preservice teachers' classroom behaviors and management skills.

The findings of this study also support the development of the VaSE system. In particular, improving the perceived ease of use of VaSE is essential. Perceived ease of use refers to how effortless an individual perceives their interactions with the VaSE to be (Huang and Liaw, 2018), which is typically related to ease of access, navigation, and interface design. Perceived ease of use is a basis for affective experience and has been confirmed to be an important measure of an individual's attitude toward VaSE (Venkatesh et al., 2003). It is also important to provide individuals with multi-channel feedback. Individuals may not readily accept text and pictures in VaSE, but they have a higher acceptance of auditory information. The visual presentation of the environment can provide corresponding auditory channels that can not only reduce the cognitive load of individuals but also prevent them from producing negative effects. It is also important to improve the realism of the experimental environment. When developing the VaSE system, it is recommended that the scene layout, object attributes, etc. are consistent with those of the real world. An environment that is highly similar to the real world will enable individuals to gain a greater sense of immersion so that they can focus on experimental operations with less external interference.

### Limitations

This study has the following limitations. The participants for this study were recruited from one university in China. This could have limited the representativeness of the study because participants from different institutions could differ from one another. Another limitation is that we did not include factors that could be related to preservice teachers' affective responses. For example, perceived usefulness and ease of use in the frame of the technology acceptance model could influence preservice teachers' affective states, and teacher efficacy could influence preservice teachers' teaching motivation. Moreover, we did not include measurements of technological pedagogical content knowledge (TPCK) in this study. In the model of TPCK, technological, pedagogical, and content knowledge are considered to be prerequisites for success when integrating technologies into the classroom. Because of the possible moderating effect on behavior, preservice teachers' affective responses will implicitly influence the operationalization of TPCK. In other words, preservice teachers' affective responses toward technologies may facilitate the effective implementation of these technologies in the classroom. Future work should use structural equations to model the multivariate effects on preservice teachers' affect and track their actual use in classrooms.

## Data availability statement

The raw data supporting the conclusions of this article will be made available by the authors, without undue reservation.

## Ethics statement

The studies involving human participants were reviewed and approved by Southwest University. The patients/participants provided their written informed consent to participate in this study. Written informed consent was obtained from the individual(s) for the publication of any identifiable images or data included in this article.

## Author contributions

TX planned and wrote the paper. LZ collected and analyzed the data. GL gave many theoretical and technical support to complete the paper. All authors contributed to the article and approved the submitted version.

## Conflict of interest

The authors declare that the research was conducted in the absence of any commercial or financial relationships that could be construed as a potential conflict of interest.

## Publisher's note

All claims expressed in this article are solely those of the authors and do not necessarily represent those of their affiliated organizations, or those of the publisher, the editors and the reviewers. Any product that may be evaluated in this article, or claim that may be made by its manufacturer, is not guaranteed or endorsed by the publisher.
