# Supplementary material for: Understanding preservice teachers' affective responses to VR-enabled scientific experiments
Source: Front Psychol. 2022 Aug 5;13:929270. doi: 10.3389/fpsyg.2022.929270 (PMC9389293; doi:10.3389/fpsyg.2022.929270)
Supplement: Supplementary file 1 [file Table_1.DOCX]

Supplementary Material

# Guidelines for Affective Experience Interviews

Note: These guidelines are translated from Chinese.

1. What do you like about the design of this virtual environment?

2. What do you dislike about the design of this virtual environment? What did you expect it to be like?

3. What do you think are the limitations of the design of this virtual environment?

4. How do you think it will make it easier for you to understand the experimental process and operation?

5. Which operations do you think are more difficult, and how do you want us to improve them?

6. What functionality do you think needs to be added to the virtual environment to get a clearer understanding of how the circuit works?

7. Do you think this is a virtual environment that can effectively help students acquire scientific knowledge? Would you recommend this environment to your friends? What changes do you think would make you more willing to recommend it to others?

8. Did you feel satisfied after the experiment was completed? And please tell me why you felt this way.

9. Did you feel a sense of accomplishment after the experiment was completed? And please tell me why you felt this way.

10. What other changes do you think would make this virtual environment more enjoyable to use?
